# Bromide of Ethyl as an Anæsthetic

**Published:** 1884-06

**Authors:** G. L. Curtis


### BROMIDE OF ETHYL AS AN ANÆSTHETIC.

BY G. L. CURTIS, D. D. S.

READ BEFORE THE FIFTH DISTRICT DENTAL SOCIETY OF THE STATE OF NEW YORK, UTICA, APRIL 8 AND 9, 1884.

I am indebted to Dr. Julian J. Chisholm, Professor of eye and ear diseases in the University of Maryland, Surgeon in charge of the Presbyterian Eye and Ear Hospital, Ophthalmic surgeon of the University Hospital, etc., of Baltimore, for valuable information in the use of bromide of ethyl; in the opinion of the writer, the most valuable anæsthetic for use in the dental profession, at least, in minor operations.

It is not without hesitation that I advance any new theory, or advocate the use of any new drug before this assembly, where the gray hairs are many, denoting wisdom and caution. But when we consider the progress of our profession, extending and advancing with perhaps greater strides than that of any other department of science, I do not hesitate to raise higher the banner that signals a new victory, and help bear the standard further to the front.

Dr. Chisholm, a most eminent surgeon, in a private communication, says: "I have given it, I suppose, 2,000 times, and use it every day, often five or six times, always most satisfactorily, and am astonished that any surgeon should be ignorant of this wonderful agent."

From this communication, and the quite extended article by Dr. C. which recently appeared in the *Medical Counselor*, advocating its use and demonstrating its advantages over chloroform and ether as an anæsthetic, particularly in minor operations such as the dentist is often called upon to perform, I was so convinced that it was the agent required to take place of the above-mentioned "stand-bys," that I resolved to give it a trial. With some trouble securing one-half ounce, all the druggists of Syracuse could boast of, on the 25th day of March, 1884, I first administered it, my patient being a robust servant-girl of seventeen years. Observing the directions of Dr. Chisholm as closely as possible, I proceeded, with the following results: Folding a thick towel in the form of a small cone with a closed apex, I placed between the folds of the towel a piece of newspaper, which made the cone nearly air-tight, then instructed my patient how to make long inspirations, and making her go through the process in advance, informed her she must do this, even though she might feel somewhat stifled, and gave her confidence by an assurance that a few inspirations would put her to sleep. Into this towel cone I poured about one drachm of the bromide of ethyl, and I immediately inverted the inhaler over the mouth and nose of my patient, holding the edges firmly down to the face. The first one or two inhalations seemed to strangle her, but the timely movement of my assistant prevented any interruption, and in about thirty seconds I removed the inhaler and was ready to operate. I found the muscles were not relaxed and the jaws firmly closed, though the conjunctiva did not respond to the touch, which delayed matters about thirty seconds. After extracting the first tooth I waited about half a minute to note the result of my drug, and then removed the second tooth. At the end of about two minutes my patient showed signs of returning consciousness, and in a half minute more awoke almost as suddenly and completely as from ordinary sleep, without any of the symptoms of nausea that generally attend the adminstration of other anæsthetics, and personally declared she felt perfectly well, and had had no knowledge of what happened during the process of ethylization.

The face of the patient was considerably flushed while under its influence, and the muscles and veins of the neck were distended. The increased action of the pulse, which was noticed, however, soon returned to its normal state.

About the same results were observed in the only other case in which I have yet administered it, but the patient, being a robust young man, recovered even more quickly than the first mentioned. Dr. Chisholm writes me that the physiological action of ethyl is not known, but the theory is defective oxidation of the blood. I have also been unable, either from our books or personal authorities, to ascertain the materia medica of this drug.

I think I should note here, as Dr. C. very aptly observes, that a recumbent posture is essential to the safe and effective administration of any anæsthetic, and that bromide of ethyl differs from others, in that while chloroform vapor freely should be diluted with atmospheric air, ethyl vapor requires the exclusion of all atmospheric air to obtain most effective results. Dr. Chisholm also says that in his earlier experiments an ignorance of this peculiarity nearly persuaded him to vote bromide of ethyl a failure, but that his later custom, to push the ethyl in its concentrated form, enables him to obtain in from twenty to sixty seconds, and to have no after-consequences of nausea or dullness of feeling.

I have given you, gentlemen, a hasty sketch of my observation in what was, so far as I was able to ascertain, the first administration of this drug in our profession, and for my own knowledge and for the general advancement of dentistry, I most respectfully solicit the experience and observations of our white-haired and more learned associates in relation to this anæsthetic.

